# Avian influenza and One Health: bridging animal and human health through integrated surveillance and vaccination

**DOI:** 10.3389/fcimb.2026.1659738

**Published:** 2026-07-13

**Authors:** Alessandro Pardini, Ruben Laurin Garnhartner, Monique Vogel, Martin F. Bachmann

**Affiliations:** 1 Department of Rheumatology and Immunology, Inselspital, University of Bern, Bern, Switzerland; 2 Department for BioMedical Research, University of Bern, Bern, Switzerland; 3 Graduate School for Cellular and Biomedical Sciences (GCB), University of Bern, Bern, Switzerland; 4 Jenner Institute, Nuffield Department of Medicine, University of Oxford, Oxford, United Kingdom

**Keywords:** avian, influenza, One Health, vaccination, virus

## Abstract

The ongoing threat of avian influenza over the past three decades represents a significant challenge for global health, livestock farming, and animal conservation. Notwithstanding the considerable strides made in research and the formulation of control strategies, the persistence of highly pathogenic strains gives rise to concerns pertaining to both animal and human health. This review examines the history and the role of the “One Health” concept in combating the disease, while first investigating the historical spread of avian influenza, its circulation in wild bird populations, the amplification and adaptation in domestic poultry, and its occasional spillover into humans, often facilitated by close contact in agricultural and live animal market settings. Historical patterns are key in understanding the dynamics of the virus and developing adequate countermeasures. Such surveillance methods and novel and rapid monitoring techniques now play a bigger role than ever. Meanwhile, improvements in genome sequencing have revolutionized our understanding of viral evolution allowing for more detailed monitoring of epidemics. Moreover, recent arguments in favor of vaccination of animals against avian influenza highlight its potential to reduce virus burden at source, reduce economic losses in agriculture, and reduce the risk of spillover to humans and other animal species, underlining its role in a comprehensive One Health approach. By integrating insights from recent technological and methodological innovations, this work advocates for a more coordinated, interdisciplinary approach to surveillance, monitoring, and vaccination. Addressing these gaps will be essential for improving outbreak preparedness and reducing the impact of this enduring threat.

## Introduction

Influenza viruses remain a persistent global health challenge, affecting humans, domestic animals, and wildlife and exerting significant socioeconomic and ecological impacts. The emergence of highly pathogenic avian influenza (HPAI) strains underscores the ongoing threat posed by zoonotic influenza, which can cross species barriers and may eventually spark a pandemic in humans (Krammer, 2018). Understanding influenza in its ecological, evolutionary, and public health contexts is therefore critical for effective prevention, surveillance, and control strategies.

This review provides a comprehensive overview of avian influenza virus (AIV) biology, evolution, and epidemiology, emphasizing the intersections between animal, human, and environmental health. It begins with a discussion of the One Health concept, highlighting its historical foundations and contemporary relevance for integrated disease management. Subsequently, the review details the virology of influenza A viruses, focusing on the molecular determinants of host specificity, transmission, and pathogenicity.

The historical emergence and propagation of HPAI, with a focus on recent H5 outbreaks, are examined to illustrate patterns of viral evolution and zoonotic risk. Building on this foundation, surveillance strategies are discussed, including the roles of domestic and wild avian populations, global initiatives, and emerging approaches to enhance early detection and data sharing. The review then addresses vaccination strategies, encompassing current approaches, limitations, and future directions for both human and animal populations.

By integrating virological, ecological, and public health perspectives, this review aims to provide a cohesive framework for understanding avian influenza in the context of One Health and to highlight the critical measures required to mitigate the impact of HPAI on animal and human populations.

## One Health concept

**Figure 1:**
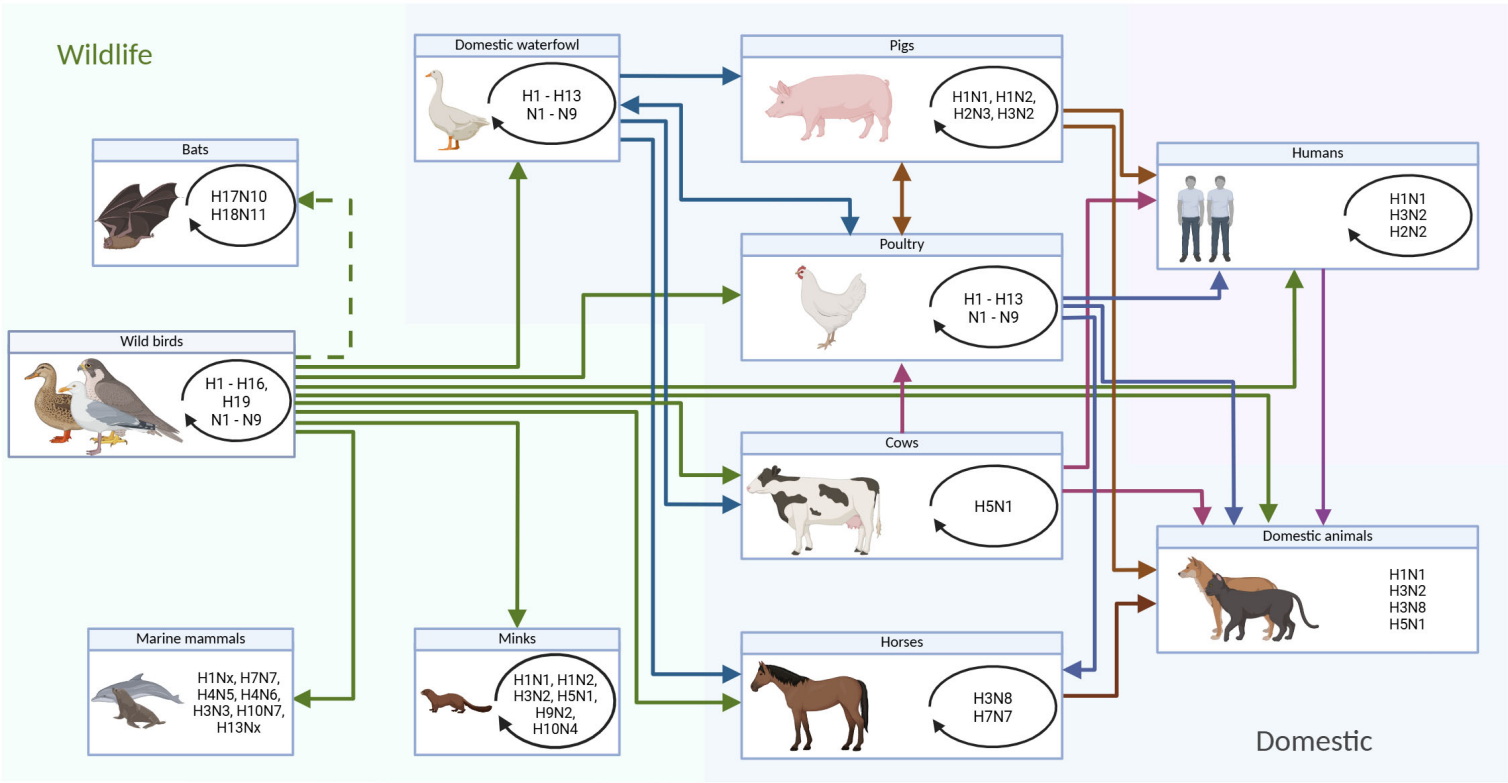
Integrating One Health for influenza control: surveillance, monitoring, and vaccination strategies. The illustration of the “One Health” concept in the context of influenza demonstrates the interdependent relationship between animal, human, and environmental health. The interconnectedness of these three domains necessitates the development of an awareness of the mutual dependencies and influencing factors to ensure sustainable and effective preparation for future challenges. The global spread of influenza not only affects human and animal health but also threatens ecosystems, as it can introduce the disease into new areas. The image was created using BioRender.com.

The notion that animal health is inextricably linked to human health is not new. As early as the 17th century, physicians recognized the role of the environment in disease transmission between humans and animals, and in the 19th century, the concepts of “zoonosis” and “One Medicine” were coined to describe the interconnectedness of human, animal, and environmental health (Kahn et al., 2008; Schultz, 2008; Evans and Leighton, 2014; Lange, 2021; Centers for Disease Control and Prevention, 2024). However, it was only in the 21st century that this perspective evolved into a comprehensive framework explicitly integrating humans, animals, and the environment. The occurrence of significant and serious outbreaks of various diseases, including avian influenza, demonstrates the detrimental impact such diseases can have on humans, animals, and the environment, causing considerable economic losses and posing a significant public health risk globally (Duan et al., 2023). This illustrates the necessity for a system that is capable of addressing all potential stages (**Figure 1**).

A One Health framework recognizes that the health of humans, animals, and the environment is closely interlinked and must be addressed collaboratively to prevent, rapidly detect, and respond to emerging disease threats such as avian influenza. In the animal health domain, this includes robust and rapid monitoring of wild and domestic animal populations, routine veterinary surveillance and early warning systems, targeted vaccination of susceptible species where appropriate, and outbreak investigations to limit virus amplification at its source; these activities help detect changes in viral prevalence and evolution before spillover occurs (Fouchier et al., 2003; Duan et al., 2023). In the human health sphere, One Health emphasizes biosecurity measures, occupational health protections for workers with animal contact, and public awareness and risk communication to reduce exposure and improve response to zoonotic threats, supported by integrated clinical and community surveillance. Environmental health considerations involve surveillance of virus persistence and spread in natural and anthropogenic habitats; monitoring how human-driven changes such as land-use conversion, habitat fragmentation, and climate change alter host–pathogen interactions; and reducing environmental pressures that facilitate pathogen emergence. By mobilizing expertise across sectors and disciplines, a One Health approach seeks to balance and optimize health outcomes for people, animals, and ecosystems in an interdependent system, recognizing that actions in one domain influence risks and resilience in the others (W.H.O T, 2017; Panel and Adisasmito, 2022; CDC, 2025).

The worldwide pandemic caused by the severe acute respiratory syndrome coronavirus 2 (SARS-CoV-2) has drawn the international community’s immediate attention to both the complexity of human–animal–environment interactions and the shortcomings in how the “One Health” concept has been operationalized in practice. In particular, the pandemic exposed deficiencies in cross-sectoral coordination, integrated surveillance, data sharing, and the consideration of environmental drivers of disease emergence. In response to these developments, the heads of the United Nations Food and Agriculture Organization (FAO), the United Nations Environment Program (UNEP), the World Health Organization (WHO), and the World Organization for Animal Health (WOAH) initiated a strengthened, science-based collaboration by establishing a multidisciplinary One Health High-Level Expert Panel (OHHLEP). The objective of this panel is to provide technical and scientific guidance on One Health, including the development of a shared conceptual framework, the identification of knowledge gaps, and the promotion of integrated approaches to surveillance, prevention, and preparedness for emerging zoonotic threats (Mettenleiter, 2023).

Adisasmito et al. defined “One Health” in 2022 as follows: “One Health is an integrated, unifying approach that aims to sustainably balance and optimize the health of people, animals, and ecosystems. It recognizes the health of humans, domestic and wild animals, plants, and the wider environment (including ecosystems) are closely linked and interdependent.” This perspective is particularly pertinent in the context of managing avian influenza, as influenza A viruses are capable of crossing species barriers and affecting both human populations and domestic or wild bird species (Krammer, 2018). Consequently, the prevention and control of highly pathogenic avian influenza require integrated strategies that consider animal reservoirs, environmental drivers, and human health risks simultaneously. A regional One Health framework therefore provides a conceptual basis for coordinated surveillance, risk assessment, and intervention strategies, highlighting the need for collaboration across disciplines and sectors in addressing emerging zoonotic threats (Panel and Adisasmito, 2022; Duan et al., 2023; Acharya and Phuyal, 2024).

## Influenza virus

Influenza viruses, which can cause acute respiratory illness and are highly contagious, continue to pose a serious threat to public health. As estimated by the World Health Organization, the annual global prevalence of clinical influenza cases in humans has reached approximately 3–5 million, with an estimated 290,000–650,000 deaths attributed to severe respiratory disease complications although other sources report even higher numbers (Iuliano, 2018; W.H.O, 2022). Nevertheless, influenza virus infection typically results in a common seasonal illness with less severe clinical manifestations, including fever, sore throat, muscular weakness, and a variety of relatively moderate respiratory symptoms. The majority of individuals recover from these symptoms in less than 2 weeks without a clear diagnosis, and a large number of infections will go unnoticed (C.D.C, 2020). However, in specific demographic subgroups, such as the elderly, children, pregnant women, and patients with immunosuppressive disorders, influenza virus infection is highly dangerous and may even be fatal (C.D.C, 2020; Chen et al., 2021).

Influenza viruses, which belong to the Orthomyxoviridae family, are negative-sense, single-stranded RNA viruses with a segmented genome. They are classified into four different genera. Influenza A and influenza B predominantly cause the disease in humans, while influenza C and influenza D have so far only been shown to infect animals (Krammer, 2018).

**Figure 2:**
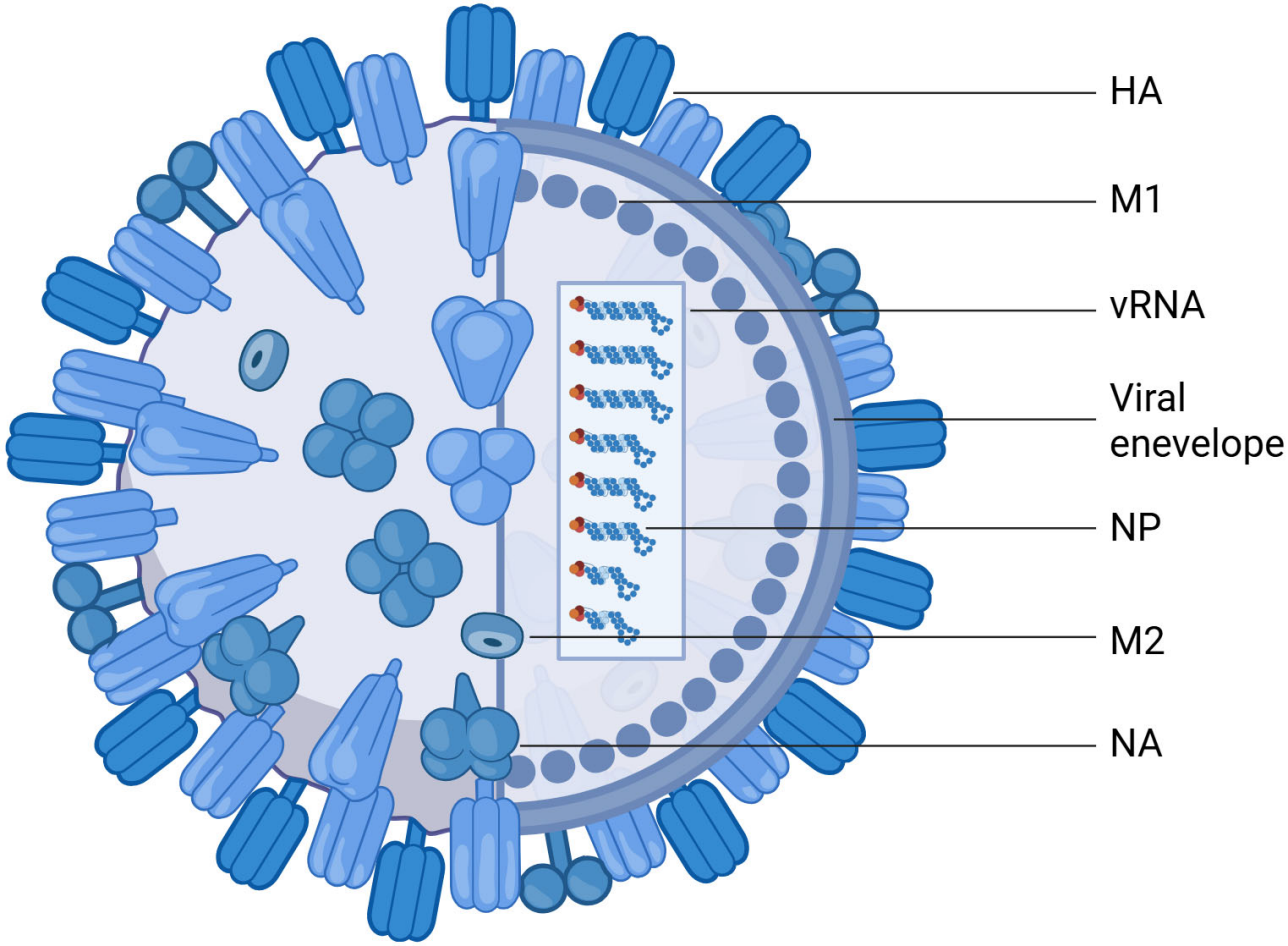
Influenza A virus. The negative-sense influenza A RNA virus image with the eight single-stranded RNA segments (vRNA) is shown. The three largest vRNA segments each encode for the RNA polymerases (PB1, PB2, and PA), which are responsible for replication within the cell through RNA synthesis. The remaining five vRNA strands encode for the haemagglutinin (HA), neuraminidase (NA), matrix proteins 1 and 2 (M1, M2), and nucleoprotein (NP) (as well as the nuclear export protein, which is not shown here). The image was created using BioRender.com.

The influenza A virus genome consists of eight negative-stranded RNA segments that encode 11 proteins (**Figure 2**). These include RNA polymerase subunits, viral nucleoprotein (NP), matrix protein (M1), membrane protein (M2), non-structural protein (NS1), and nuclear export protein. The two major surface glycoproteins, haemagglutinin (HA) and neuraminidase (NA), perform important functions in the life cycle of the virus (Krammer, 2018). From an immunological point of view, NA is regarded as immune subdominant to HA, and anti-NA antibodies are not routinely assessed to predict protection against disease. Notably, the virus’s surface contains a greater number of HA molecules than NA, which results in elevated levels of anti-HA antibodies following natural infection (Uno and Ross, 2024), and HA is also the molecule responsible for cellular attachment and infection.

Indeed, HA plays a pivotal role in the viral infection process, whereby it binds to receptors containing glycans with terminal sialic acids on the cell surface, facilitating the fusion of the viral and host endosomal membranes and enabling the transfer of viral nucleic acid into the host cell cytoplasm (Stevens, 1979; Huang, 2023). The influenza virus HA glycoprotein is a trimer with two distinct structural domains—HA1 and HA2. HA2 is a triple-stranded coiled-coil of alpha helices with more conserved regions, while HA1 is a globular domain composed of antiparallel beta sheets. The globular domain HA1, located at the top of the stem, contains the receptor binding site and the variable antigenic determinants, which are highly prone to mutations and constitute the primary targets for most neutralizing antibodies (Wilson et al., 1981; Webster et al., 1982).

In avian influenza, the viral HA1 protein has been demonstrated to bind directly to α2,3-linked sialic acid cell membrane proteins in birds, facilitating cell entry in intestinal and respiratory epithelium. In contrast, human influenza viruses predominantly recognize α2,6-linked sialic acid, found on the human upper respiratory tract (Krammer, 2018; Huang, 2023). The alteration in receptor specificity from α2,3-linked sialic acid (avian) to α2,6-linked sialic acid represents a significant challenge for influenza viruses to jump species, although it can be overcome with as few as two amino acid substitutions in the HA receptor-binding site, allowing HA to accommodate the distinct conformations of the receptors and facilitating zoonotic spillover. However, such adaptive mutations often reduce viral fitness, thereby limiting efficient infection and sustained human-to-human transmission (Long et al., 2019).

The NA glycoprotein structurally forms a tetramer with a catalytic head, stalk, transmembrane region, and a cytoplasmic tail (McAuley et al., 2019). It contributes critically to the viral life cycle by cleaving terminal sialic acids from host cell receptors and newly assembled virions, thereby facilitating virion release and preventing aggregation caused by HA binding (Palese et al., 1974). Beyond its role in viral egress, NA counteracts the host mucus barrier via its sialidase activity and can contribute to early stages of infection by mediating interactions with sialoglycoproteins on the cell surface (Yang, 2016; Guo, 2018).

The primary target for neutralizing antibodies is the major antigen on the viral surface, namely, the HA. HA plays a critical role in viral attachment to host receptors, enabling the virus to enter the host cell via endocytosis and subsequent membrane fusion. Consequently, the HA represents a valuable target for both therapeutic and vaccine approaches (Isakova-Sivak and Rudenko, 2024). Although 19 avian and mammalian HA serotypes are known, only three (H1, H2, and H3) have been adapted to humans (Chen, 2014). In contrast, NA is not yet routinely exploited as a primary vaccine antigen, despite accumulating evidence that NA-specific antibodies can confer protection and may contribute to broader and more durable immunity (Cortés et al., 2024).

Influenza viruses evolve primarily through two mechanisms: antigenic drift and antigenic shift. Antigenic drift involves the gradual accumulation of point mutations in viral genes, most notably in the HA and NA, leading to incremental changes in antigenicity over time (Webster et al., 1982; Kim et al., 2018). Mutations affecting antigenic regions of mostly HA but also NA can confer a selective advantage by enabling escape from antibody-mediated immunity, allowing viruses to persist in populations despite prior infection or vaccination and necessitating regular updates of influenza vaccines. The dynamics of antigenic drift are influenced by host immunity, the introduction of susceptible individuals, viral fitness, and seasonal factors, contributing to the continuous evolution of circulating strains (Webster et al., 1982; Axelsen et al., 2014; Kim et al., 2018; Sasaki et al., 2022). Studies of H3N2 (Hong Kong) influenza viruses have demonstrated that multiple antigenic variants can co-circulate during epidemic periods, highlighting the complexity of HA evolution (Both et al., 1983).

In contrast, antigenic shift is characterized by the acquisition of a novel HA gene through genomic reassortment, often occurring during co-infection of a host with influenza A viruses of different origins, including animal strains. Antigenic shift results in abrupt changes in antigenic properties and is typically associated with influenza A pandemics, frequently leading to the displacement or extinction of previously circulating strains (Webster et al., 1982; Krammer, 2018). For vaccine design, the fact that exchanging HA only on influenza viruses is sufficient to potentially cause a pandemic is highly noteworthy. Indeed, it demonstrates that T cells specific against all the other shared proteins are apparently not able to mediate antiviral protection. Hence, the development of vaccines based on T cells against the influenza virus may be a dubious task.

## Avian influenza A viruses: evolution, spread, and public health implications

Avian influenza A viruses are further classified into subtypes based on the antigenic properties of these surface proteins, resulting in a variety of HA and NA combinations. Of the 19 known HA subtypes and 11 NA subtypes, 17 HA and 9 NA subtypes are commonly found as low pathogenic avian influenza (LPAI) viruses in wild waterfowl worldwide (Halbherr, 2015). However, in poultry, only H5 and H7 have undergone a change from LPAI to HPAI strains through the acquisition of changes at the HA cleavage site, thereby enabling systemic infection that results in damage to vital organs and tissues, ultimately leading to bird death (Monne, 2014; Xie, 2023).

### History of avian influenza

The relevance of avian influenza was recognized long before the development of modern techniques for analyzing viral evolution. In 1878, a serious poultry disease swept through Piemont (IT), causing significant losses of chickens, guinea fowl, ducks, and geese. Eduardo Perroncito referred to this outbreak as “bird cholera” or “epizootic typhus,” noting its similarity to earlier recorded epizootics across Europe. This outbreak is considered one of the first reports of avian influenza in Europe. Perroncito emphasized the agricultural and societal impact of such epizootics, foreshadowing contemporary concerns regarding the economic, ecological, and public health consequences of avian influenza outbreaks (Perroncito, 1878; Lupiani and Reddy, 2009). This early observation highlights that the capacity of avian influenza viruses to spread rapidly and cause severe disease has been recognized for over a century, underscoring the long-standing need for surveillance and control strategies.

### Role of migratory birds and evolutionary changes

**Figure 3:**
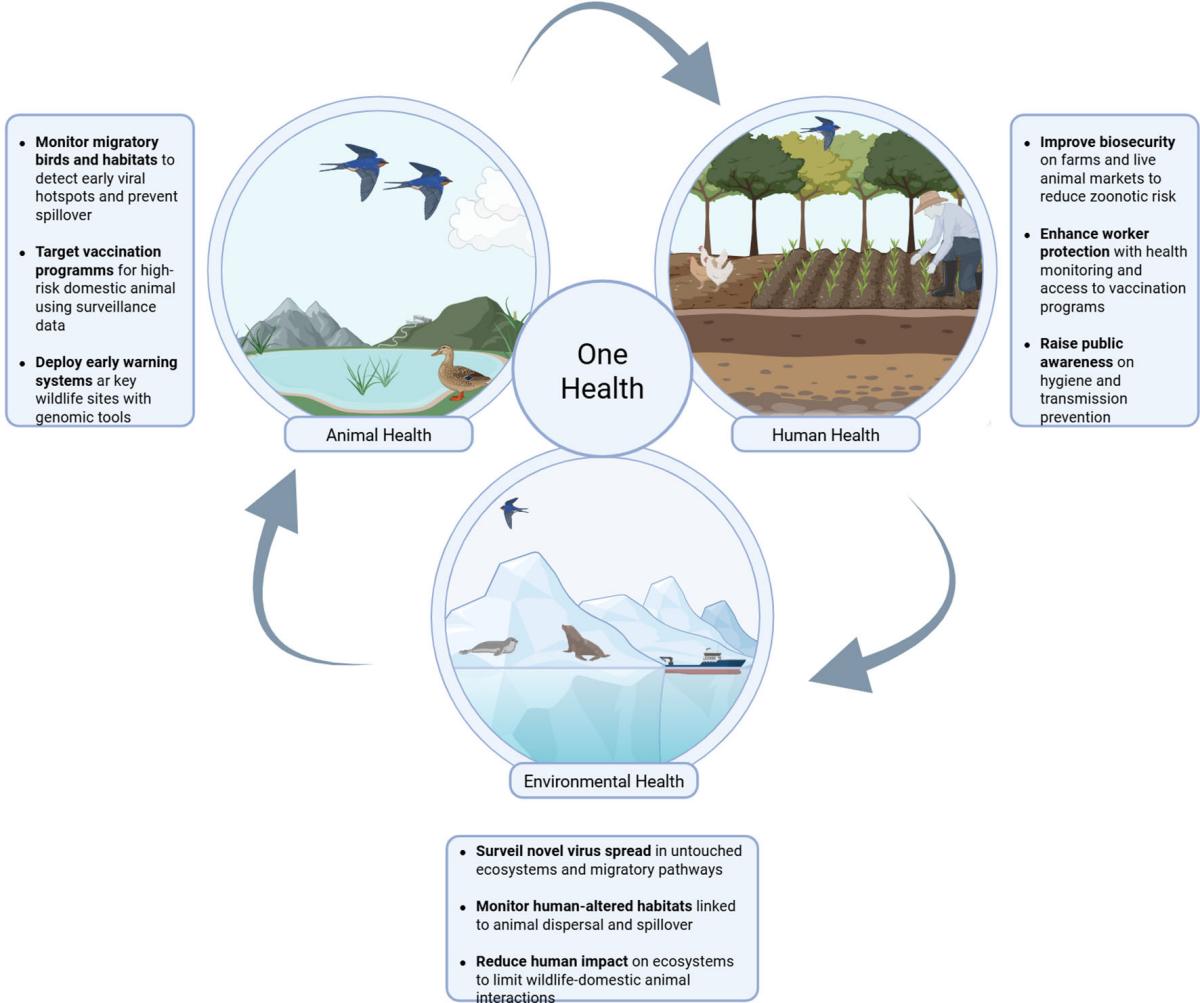
Ecological and cross-species interfaces of influenza. Shown here is the predicted transmission of the avian influenza viruses. Wild birds act as reservoirs for the virus, facilitating infection of a wide range of animals through various transmission routes. Some animals, including wild birds or pigs, are capable of transmitting and reassembling the virus within the species, as illustrated by the black circle within the boxes. It is crucial to understand the range of animals that can be infected by avian influenza and the potential risks this poses to different animals and humans. The illustration was adapted from Graziosi et al. (Long et al., 2019; Graziosi et al., 2024; Peacock, 2024) The image was created using BioRender.com.

Phylogenetic analysis of influenza A virus RNA segments from different hosts and regions reveals that migratory birds, especially waterfowl, play a crucial role in the maintenance and spread of influenza A viruses (Webster et al., 1992; Gils, 2007). These viruses have evolved into host-specific lineages, with avian strains contributing to mammalian influenza and occasional gene exchange leading to pandemics. Current influenza strains in humans, pigs, horses, minks, marine mammals, and a wide range of domestic birds are likely to have originated from avian reservoirs, with possible viral genetic exchange involving cows and other animals, and ongoing research aims to understand these dynamics to prevent future outbreaks (**Figure 3**) (Webster et al., 1992; Olsen, 2006; Taylor, 2023; Caserta, 2024; Eisfeld, 2024). Avian species, particularly waterfowl and shorebirds, serve as natural reservoirs for AIVs, providing environments conducive to virus growth, mutation, and, on occasion, transmission to other species. These viral transfers between birds and other animals, including humans, underscore the persistent risk of influenza viruses that could potentially trigger future pandemics. The dissemination of AIVs is, therefore, not merely a concern for animal populations; it constitutes a significant public health issue and emphasizes the importance of addressing avian influenza within a One Health framework that integrates human, animal, and environmental health to mitigate the risk of further pandemics (Blagodatski, 2021). Given the damage inflicted on the world by a pandemic caused by a relatively mild SARS-CoV-2 virus, the consequences of a pandemic caused by a highly pathogenic influenza virus cannot be underestimated.

### Environmental drivers

The influence of climate processes on the ecology of birds and other wildlife as well as on viral evolution is subject to variation across both spatial and temporal scales. Indirect consequences can be anticipated, specifically those resulting from the impact of climate change on agro-ecosystems associated with duck and crop production, as well as alterations in the location of domestic and wild waterfowl interaction sites (Gilbert et al., 2008). These effects encompass a range of changes, from long-term shifts in the distribution, migration patterns, and population density of bird species, to immediate impacts on the viability of the virus in the environment (Fusaro, 2019; Prosser et al., 2023). The dissemination and evolution of diseases depend on these ecological interactions among wild and domestic birds, as well as their surrounding environments (Prosser et al., 2023). In turn, the conservation of habitats could serve to reduce the probability of avian influenza outbreaks by minimizing the degree of overlap between wild and domestic birds, the preservation of natural ecosystems that support healthy wildlife populations, and mitigation of the factors that drive viral outbreak events.

The evolutionary dynamics of influenza viruses, driven by viral interspecies gene exchanges and the role of avian reservoirs, have long been a significant factor influencing the emergence and spread of new viral strains (Taubenberger and Kash, 2010). The capacity of AIVs to adapt and transmit between diverse hosts establishes potential pathways for zoonotic infections, which represents a distinctive challenge in this regard. This emphasizes the necessity of focusing on AIVs as a significant area of concern in both public health and animal health. It is imperative to gain an understanding of the evolutionary and transmission dynamics of these viruses across species if effective strategies for the prevention and mitigation of future outbreaks and new pandemics are to be developed. From an evolutionary standpoint, this perspective provides crucial insights into the continued necessity of permanent monitoring and robust preparedness strategies for the management of both current and emerging threats. The lessons learned from past influenza pandemics underscore the significance of surveillance, particularly in the context of avian reservoirs, to diminish the probability of future global health crises within a multilateral engagement.

## The history of global dissemination and propagation of the influenza A virus H5

### H5N1 prior to human infection

HPAI viruses of the H5 subtype were recognized in poultry decades before their emergence as zoonotic pathogens. As mentioned, the first confirmed outbreak of HPAI was reported in domestic poultry and wild birds in Italy in 1878 (Perroncito, 1878), although the viral etiology was not established at the time. Following the identification of influenza A viruses in the early 20th century, H5 and H7 subtypes were subsequently shown to be uniquely associated with high pathogenicity in poultry, primarily due to the acquisition of a polybasic cleavage site at the HA cleavage region, which facilitates enhanced viral activation and systemic infection (Webster et al., 1992; Steinhauer, 1999). Throughout the latter half of the 20th century, H5 avian influenza viruses circulated predominantly as low-pathogenic strains in wild aquatic birds, while sporadic spillover into domestic poultry occasionally resulted in the emergence of HPAI through mutation during replication in gallinaceous hosts (Webster et al., 1992; Olsen, 2006). The first outbreak of an HPAI H5N1 virus strain was recorded in Scotland in 1959, with several further outbreaks following, causing substantial economic losses, but with no confirmed human infections. During this period, H5 viruses were therefore regarded largely as an animal health concern, with limited attention paid to their zoonotic potential (Pereira et al., 1965; Halvorson, 2009; Lycett et al., 2019).

### H5N1 in Hong Kong (1997)

**Figure 4:**
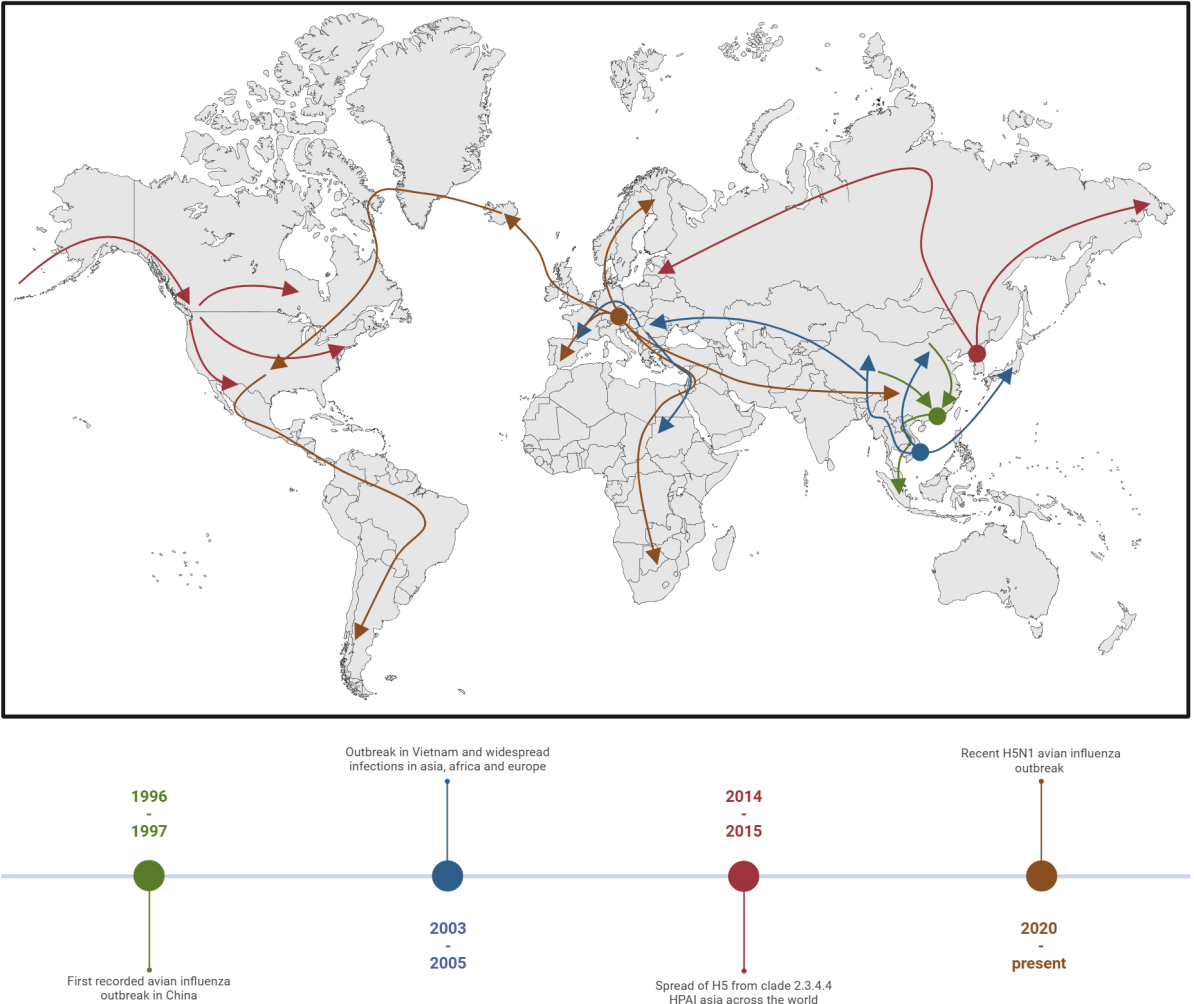
Global dynamics and historical timeline of avian influenza spread. The illustration depicts a selection of the most prevalent avian influenza pandemics over the last 30 years. The dots and arrows on the map indicate the potential origin and the initial dissemination of the disease through avian vectors. The timeline commences with the initial documented instance of human infection by avian influenza in 1996 in Hong Kong. Additionally, the pandemics in Vietnam between 2003 and 2005, and subsequently in Europe, as well as the initial dissemination of the HPAI clade 2.3.4.4b from Asia to Europe and America, are also illustrated. Ultimately, the pandemic of the HPAI H5 virus is now detectable globally (Lycett, 1979; United States Department of Agriculture, 2016; Charostad, 2023; Nielsen, 2023). It is crucial to emphasize that the arrows do not signify a concluded trajectory, but rather possible initial dissemination of the virus. The virus is in a constant state of propagation and mutation. The image was created using BioRender.com.

In 1997, a notable incident in Hong Kong resulted in 18 cases of infection with the HPAI H5N1 virus, with six deaths attributed to the infection (**Figure 4**). This marked the first documented occurrence of a highly pathogenic H5N1 strain infecting humans, attracting global attention (Charostad, 2023). Although transmission was initially thought to have occurred via chicken-to-human contact, concerns were subsequently raised about the potential for efficient human-to-human transmission, which could signal the emergence of another influenza pandemic. Epidemiological studies later confirmed instances of human-to-human transmission, though its efficiency remained very low (Chan, 1997). Further analysis of the isolated nucleotide sequences revealed that the human virus A/Hong Kong/156/97 was genetically closely related to the avian strain A/chicken/Hong Kong/258/97, thus emphasizing the link between human infections and avian reservoirs (Claas, 1998).

Molecular epidemiological studies have identified the source of the lethal H5N1 strain as the HA gene of the A/goose/Guangdong/1/96(H5N1) virus, which was isolated from a goose in China during a 1996 outbreak. The gene in question exhibited high similarities to the H5N1 viruses identified in humans in Hong Kong in 1997; however, other gene segments, particularly the NA gene, displayed notable differences (Subbarao, 1979). Given that poultry farms in Guangdong supply poultry to Hong Kong, it is postulated that the HA genes in the outbreak may have originated from a virus that is similar to the Guangdong strain. In contrast, the other gene segments are thought to have derived from different progenitors, indicating a genetic shift (Subbarao, 1979; Chan, 1997; Xu et al., 1999). This Guangdong lineage has since played a crucial role in the global epidemiology of avian influenza. All currently circulating highly pathogenic H5N1 viruses, which are now endemic in various regions worldwide, can be traced back to the A/goose/Guangdong/1/96 strain (Krammer and Schultz-Cherry, 2023).

### H5N1 in Asia (2003–2004)

The largest outbreak of HPAI H5N1 affected poultry across eight Asian countries, including Vietnam, Thailand, Indonesia, and China. Domestic ducks played a pivotal role in the dispersal of the virus to terrestrial poultry, which was a crucial expansion in the host range of H5N1 from geese to ducks (Sims, 2005; Tiensin, 2005). While the number of human infections remained relatively low, the mortality rate in Vietnam and Thailand reached 70% (Trampuz et al., 2004). The virus was initially identified in poultry in mid-2003 in Vietnam, Indonesia, and Thailand, subsequently spreading rapidly across the region. Phylogenetic analyses revealed the presence of multiple genetic reassortants in Hong Kong and mainland China, all of which exhibited HA genes that were similar to those of the A/goose/Guangdong/1/96 strain. It is noteworthy that nine distinct genotypes were identified in domestic ducks, which differed from those observed in poultry. This finding highlights the pivotal role that ducks and land-based poultry have played in the evolution of the virus, and underscores the contribution of wild birds to the virus’s widespread dissemination (Horimoto and Kawaoka, 2005; Sims, 2005).

### Global spread of H5N1 and H5N8

The global outbreak of avian influenza in 2014–2015 was driven by a combination of environmental factors and migratory bird movements, primarily involving the H5N1 and H5N8 strains (Lycett, 1979). Recent studies have demonstrated that migratory birds have been pivotal in the dissemination of HPAI, particularly clade 2.3.4.4 of H5. This clade was previously confined to eastern Asia from 2004 to 2012. The findings suggest that the seasonal movements of these birds have likely facilitated the global spread of the virus, including the 2014–2015 outbreaks in Europe and North America. This is contrary to the hypothesis that the live poultry trade was the primary vector for the virus’s global dissemination (Lycett, 1979; Yang, 2024). Further supporting this, the H5N8 virus followed specific bird migration routes, originating in South Korea during the winter of 2013–2014 and then spread from South Korea to Japan, North America, and Europe, resulting in widespread outbreaks between autumn 2014 and spring 2015.

Phylogenetic analyses revealed two major migratory routes: one from the East Asia coast and the Korean peninsula to Europe, and another via the Bering Strait to North America (Lycett, 1979). While migratory birds were crucial in the spatial spread of the virus, novel studies revealed that different environmental factors also played a significant role in shaping the outbreak patterns. A comparative analysis of the H5N1 virus between the years 2005 and 2019 revealed that the majority of epidemics occurred in Africa, followed by Asia and Europe, with the American continent being less affected. Seven environmental variables were identified as potential contributors to the outbreaks, including protective factors such as water area distance, road distance, railroad distance, and wind speed, among others. However, analysis revealed that air temperature was a significant risk factor, indicating a greater likelihood of outbreaks occurring in warmer environments (Chen, 2022). These global patterns were exemplified by the HPAI H5N1 outbreak in the United States during 2014–2015, which resulted in the loss of approximately 43 million egg-laying pullet hens and 7.4 million turkeys, in addition to a small number of mixed poultry flocks. These animals were either culled as part of the response or died as a consequence of the disease. This outbreak represents one of the most significant animal health incidents in American history, representing the largest HPAI outbreak ever documented in the country (Agriculture, V. S. S. P. A. R. S. & S, P. H. I, 2016). Despite the large scale of the outbreak and numerous human exposures to HPAI H5-infected birds, no human infections were confirmed, indicating a low risk of zoonotic transmission in the United States up to now (Arriola, 2014).

From 16 September to 8 December 2021, HPAI was recorded a total of 867 times in 27 EU/EEA countries and the United Kingdom, affecting poultry, wild birds, and captive birds. A majority of the reports came from Germany, the Netherlands, and the United Kingdom. It was already anticipated that the cases of HPAI would pose a significant risk for the poultry industry in Europe (Adlhoch, 2021).

The results of genomic analysis indicate that the viruses described during this reporting period belong to clade 2.3.4.4b. Novel reassorted HPAI A(H5N1) viruses have been introduced in Northern, Central, Southern, and Eastern Europe since October 2021. Some of the characterized HPAI A(H5N1) viruses found in Sweden, Germany, Poland, and the United Kingdom are related to the viruses circulating in Europe since October 2020 (Adlhoch, 2021). Interestingly, mink farms were among the first settings in which clade 2.3.4.4b HPAI A(H5N1) virus infections were reported (Agüero, 2022). Further genetic sequencing and RT-PCR testing confirmed that viruses from the 2.3.4.4 clade are particularly pathogenic, likely contributing to the rapid spread and severity of the outbreak (Günther, 2022).

In northern Europe, minks are still farmed for their fur in large farms. The identified virus from minks had a high similarity to an avian influenza strain that was present across northern Europe. Furthermore, the strain identified in minks exhibited a notable mutation in the PB2 gene (T271A), which enhances the virus’s polymerase activity in mammalian cells, potentially increasing the risk of adaptation to mammalian hosts and public health concern (Agüero, 2022). In addition, HPAI A(H5N1) has also been found in wildlife in Sweden, Estonia, and Finland, and some of the strains described so far have changes that are associated with increased virulence and replication in mammals (Adlhoch, 2021). Together with recent evidence for mutations in HA that increase binding to mammalian receptors, these findings indicate that the 2021−2022 H5 HPAI resurgence was not only exceptional in scale but also characterized by episodic evolutionary changes that may increase the zoonotic potential of contemporary H5 (Xie, 2023).

Notably, in December 2021, a series of avian mortalities due to HPAI H5N1 occurred on an exhibition farm in St. John’s, a city situated on Canada’s Atlantic coast. This marked the first report of HPAI to North America since June 2015, when the virus was transmitted by wild birds over the Bering Strait to the Pacific coastlines of Canada and the USA via the Pacific Flyway, one of the primary avian migratory routes (Caliendo, 2022). It was not only migratory birds that were infected; it was also reported that HPAI infection was the leading cause of mortality in gray seals in New England, which occurred simultaneously with avian infections. A study found through phylogenetic analysis that the virus genome from avian and seal samples was closely related, suggesting the possibility of an adaptation pathway of the virus from birds to seals (Charostad, 2023).

### Introduction of HPAI to South America

The spread of HPAI A(H5N1) to the USA in 2015 marked a key milestone in the virus’s global dispersal and its first confirmed introduction to South America, a continent whose relative geographic isolation had historically protected poultry industries and fragile coastal ecosystems from epidemics observed in North America (Leguia, 2023). However, clade 2.3.4.4b is spreading more rapidly, killing large numbers of birds, infecting mammals, and entering countries such as Peru, Ecuador, and other South American countries that have been HPAI-free for decades (Bruno, 2023; Leguia, 2023). The introduction of the novel HPAI into South America was presumably through migratory birds travelling south during the winter months, with multiple possible transmission routes. These birds share their habitats together with marine mammals such as sea lions and dolphins that can be infected through close contact with infected birds. This is very concerning as these affected areas do not have prior exposure and knowledge in managing HPAI viruses in wildlife and domestic poultry (Leguia, 2023). In addition, marine mammals will not have a preexisting immunity to these viruses. Kandeil et al. showed in their study in 2023 that H5Nx reassortment and phenotypic diversification have the potential to happen quite fast even in natural systems, further increasing the global distribution potential of this virus (Kandeil, 2023). Novel insight shows that the virus of clade 2.3.4.4b displays a significant level of genetic elasticity with regard to reassortment with other viruses, which has resulted in the emergence of a diverse range of over 50 different genotypes with the HA segment. Notably, the virus now threatens not only wild and domestic bird populations but has also impacted domestic livestock, including dairy cattle (Abdelwhab and Beer, 2024).

### Emergence and cross-species transmission of H5N1: roles for the biodiversity and One Health approach

Recently, a critical reassortment event in a yet unidentified host species resulted in the emergence of the H5N1 genotype B3.13, which initially circulated in wild birds and mammals before infecting dairy cattle. The virus was able to rapidly transmit between cows and other species, including wild birds and mammals. Intraspecies transmission between cows and between different animals such as birds and human personnel in the vicinity of the farms fueled the rapid transmission of the virus across different farms (Caserta, 2024). The history and recent spread of influenza viruses demonstrate the intricate interrelationship between diverse hosts, encompassing wild and domestic animals, as well as humans. This dynamic interaction emphasizes the significance of both long-standing and newly emerging strains of influenza, which continue to impact biodiversity, animal health, and human populations. These cross-species infections not only present a risk to the animals themselves but can also disrupt ecosystems and biodiversity as affected wild bird populations decline (Leguia, 2023).

The One Health approach, which acknowledges the interconnectivity of human, animal, and environmental health, is vital for comprehending and combating the influenza virus (W.H.O T, 2017). This framework proposed by the WHO highlights the necessity for integrated surveillance and monitoring of influenza across species, as early detection and monitoring can assist in mitigating the virus’s spread and preventing future outbreaks. By maintaining a vigilant observation of influenza in both wildlife and domestic settings, health authorities and researchers can enhance the capacity to predict, respond to, and control outbreaks, thereby reducing risks for all species involved.

## Surveillance and monitoring of the avian influenza virus

As discussed before, the propagation of the avian influenza virus depends on a multitude of variables. Over the past decades, numerous containment strategies have been devised in response to each avian influenza pandemic. It is crucial to enhance timely surveillance for both veterinary and human viruses to ensure pandemic preparedness. At the global and regional levels, the surveillance network has expanded considerably, becoming an important strategy for mitigating avian influenza. Key components include the effective and real-time surveillance and data collection, which both strengthen the scope and accuracy of monitoring efforts. Although the overarching goal of monitoring avian influenza remains constant, the specific approaches and models employed vary, reflecting regional or national distinctions in the frameworks and applications of surveillance systems (Duan et al., 2023).

Effective monitoring techniques are required to assess and predict the transmission of the virus and evaluate the threats to human, poultry, and wildlife health. The monitoring of both domestic and wild animals, with a particular focus on migratory avian species, represents a fundamental element of an effective data collection and analysis strategy. It is vital to gain a comprehensive understanding of the specific avian species that require the most observation and have the potential to exert the most significant influence on the global surveillance and distribution of the virus (Yang, 2024). Animal surveillance thus plays a key role in the timely detection of viruses that may pose a risk to humans (Lycett, 1979; Fouchier et al., 2003; Günther, 2024).

Surveillance strategies for avian influenza differ substantially between wild bird populations and domestic poultry systems, reflecting their distinct epidemiological roles. Surveillance in wild birds, particularly migratory waterfowl and shorebirds, primarily serves as an early warning system, enabling the detection of novel strains, monitoring viral evolution, and identifying long-distance dissemination pathways. Such surveillance is often based on targeted active sampling and passive reporting of mortality events but remains logistically challenging and unevenly distributed across regions (Poulson and Brown, 2020; Blagodatski, 2021; EFSA, A et al., 2023). In contrast, surveillance in domestic poultry is more structured and intervention-oriented, integrating routine testing, outbreak investigations, and biosecurity measures at the farm level to enable rapid containment and limit economic losses (EFSA, A et al., 2023). Effective control of avian influenza therefore relies on the integration of wildlife and agricultural surveillance systems, as each addresses complementary aspects of virus emergence, amplification, and transmission.

One of the most effective methods for the detection of HPAI in migratory and domestic bird populations is the passive surveillance and sampling of deceased or diseased birds with a real-time reverse transcription polymerase chain reaction assay, followed by the determination of the viral pathotype as HPAI or LPAI, by further investigating the positive samples by sequence analysis (Hesterberg, 2009; James, 2022). It is thus imperative that animal testing be conducted regularly and that the results thereof, along with genomic sequencing and rapid and unrestricted access to epidemiological data, be made available for open access. The development and implementation of more rapid and innovative monitoring solutions are imperative. Such systems might include decentralized reporting systems that can rapidly analyze and disseminate crucial information to adequately respond to newly emerging threats (Branda et al., 2024). To this end, animal health and human diagnostic centers may work together in less accessible areas.

Together, these surveillance approaches enable the timely collection and integration of animal influenza data, supporting risk assessment and coordinated responses across animal and human health sectors in line with a One Health framework (Fouchier et al., 2003).

### Global surveillance initiatives

One proven system is the Global Influenza Surveillance and Response System (GISRS), which was established in 1952 and encompasses a global network of laboratories that monitor the spread of influenza and provide the WHO and member states with crucial virus information (W.H.O, 2020). In response to restricted access to data on human H5N1 virus fatalities during the 2006 outbreak, the Global Initiative on Sharing All Influenza Data (GISAID) was established by members of GISRS in collaboration with governmental and non-governmental health agencies and influenza researchers. Officially launched in 2008, GISAID operates as an independent data-sharing platform that supports GISRS through its EpiFlu™ database, promoting the rapid exchange of influenza data while ensuring data ownership, contributor recognition, and controlled access under its Database Access Agreement. The platform hosts genetic sequence, clinical, and epidemiological data for human influenza viruses, as well as regional and species-specific data for avian and other animal viruses (Shu and McCauley, 2017).

Building on the success of global initiatives like GISRS and GISAID, innovative surveillance methods have emerged to address other public health threats. For example, the high proportion of asymptomatic infections in humans that contribute to SARS-CoV-2 transmission has led to the conclusion that wastewater monitoring data are especially useful in the management of the COVID-19 pandemic. The utilization of SARS-CoV-2 data derived from wastewater testing has been linked to the success of public health initiatives (Honein et al., 2024). In light of the aforementioned developments, the CDC and its partners have initiated an expansion of wastewater surveillance activities in response to the outbreak of the highly pathogenic avian influenza A (H5N1) virus in cattle and poultry in the United States, as well as the subsequent emergence of human cases (Louis, 2024). Such surveillance initiatives benefit from collaboration with established expert networks focused on avian influenza. The OFFLU network, which was established in 2005 by the Food and Agriculture Organization of the United Nations and the World Organization for Animal Health, is such a consortium of avian influenza experts. The objectives of the OFFLU network are threefold: firstly, to research avian influenza; secondly, to provide veterinary advice to member nations; and thirdly, to interact with the WHO animal influenza network (Edwards, 2006). The OFFLU initiative still plays a pivotal role in providing crucial data from the animal health community and the biannual WHO vaccine composition meeting, which is instrumental in contextualizing zoonotic avian and swine influenza outbreaks in humans (WOAH, F, 2025).

### Challenges and future directions

Despite the implementation of novel surveillance approaches, including expanded active sampling, environmental surveillance, and high-throughput sequencing, these advances have not been accompanied by a corresponding reduction in surveillance biases (Duan et al., 2023). These discrepancies are more evident in regions with lower surveillance effort, such as Siberia, Africa, and South America, but are also observed in densely populated and comparatively well-sampled regions such as Europe (Fusaro, 2019; Blagodatski, 2021; Bruno, 2023). Even in these regions, tracking of specific bird species remains challenging and incomplete, showing how much data would be needed to precisely determine specific bird movement (Bernard et al., 2021; Yang, 2024).

To address these challenges, the rapid detection and accurate categorization of novel potential emerging influenza viruses are paramount. Based on the most recent research findings, there is a pressing need for improved international collaboration in research, surveillance, and control across a multitude of levels, extending from small regional locations across countries and continents. Especially, under sampled regions can have great significance for the processing of the virus, so greater caution is required and all regions must be included. It is also essential that the transfer of new findings from research and political initiatives can be exchanged swiftly and efficiently on international platforms, to facilitate the prompt and effective evaluation of potential threats to humans, animals, and the environment (Bernard et al., 2021; Ciminski et al., 2023; Prosser et al., 2023).

### Biosecurity

Biosecurity constitutes a cornerstone of AIV prevention and control, particularly in poultry production systems where the risk of virus introduction and amplification is highest. Effective biosecurity measures aim to reduce both the likelihood of AIV introduction and subsequent within-farm spread by limiting contact between domestic poultry and wild birds, controlling human and vehicle movement, enforcing sanitation and disinfection protocols, and ensuring appropriate management of food, water, and waste. In regions affected by HPAI, enhanced biosecurity has been shown to significantly reduce outbreak incidence and transmission between farms, often representing the most immediate and cost-effective mitigation strategy in the absence of vaccination. Moreover, biosecurity is a key component of One Health-oriented preparedness, as it not only protects animal health and agricultural livelihoods but also reduces opportunities for zoonotic spillover and viral adaptation at the human–animal interface (Whitlow, 2025). Consequently, international organizations such as the FAO and WOAH emphasize biosecurity as a fundamental element of integrated AIV surveillance, control, and eradication programs (F.A.O, 2011; World Organisation for Animal Health (WOAH), 2024a).

## Influenza vaccines

Seasonal influenza remains a significant public health challenge, while a pandemic strain could impose an even greater burden on global health and the economy (Osterholm et al., 2012). The development of vaccines for influenza A and B viruses commenced in the 1940s. The initial vaccines were produced in embryonated chicken eggs and consisted of coarsely purified, inactivated viruses treated with formalin and phenylmercuric nitrate (Francis et al., 1945). The influenza vaccination has been demonstrated to reduce the severity of illness in individuals who contract the virus despite being vaccinated. This is particularly evident in the reduction of the risk of intensive care unit (ICU) admission and death among hospitalized adults, as well as the lowering of the incidence of fever in children with confirmed influenza, which serves to highlight the benefits of the vaccine across both severe and milder cases of the disease. Given their established safety and still moderate efficacy, current influenza vaccines continue to play a vital role in reducing illness and its impacts (Osterholm et al., 2012; Ferdinands, 2021; Taaffe, 2024). Thus, since 2010, the CDC and the Advisory Committee on Immunization Practices (ACIP) have recommended routine influenza vaccination for all persons aged ≥6 months who do not have contraindications (Grohskopf et al., 2024). The importance of maintaining preparedness for a potential influenza pandemic remains evident. Without a sustained influenza vaccine infrastructure, as is currently employed for seasonal influenza, manufacturing capacity would likely be insufficient, particularly given that most licensed vaccines are still produced using egg-based systems. Hence, modernization of vaccine production seems an easy and affordable measure and yet is rarely tried, in part due to resistance within the industry.

### Vaccine technologies and approaches

Influenza vaccines, currently used or under development, encompass a variety of approaches, including inactivated whole virus vaccines, split-virion vaccines, subunit vaccines, virosome-based vaccines, live attenuated vaccines, and recombinant live viral vectored vaccines (Wei, 2020; Chen et al., 2021). For humans, current, registered influenza virus vaccines are produced using egg-based, cell-based, or recombinant techniques, with inactivated egg-based vaccines remaining by the far most widely used (W.H.O, 2022). Guidelines were therefore developed that encompass the quality, regulatory, non-clinical, and clinical elements of influenza vaccine development (Committee for Medicinal Products for Human Use, 2016). For animals, on the other hand, vaccines must adhere to the Manual of Diagnostic Tests and Vaccines for Terrestrial Animals, and include, among others, an RNA replicon vaccine against H5N1 avian influenza virus subtype and inactivated whole virus vaccines against canine influenza (Ellis et al., 2022; World Organisation for Animal Health (WOAH), 2024b; Stettler et al., 2025).

The majority of influenza vaccines have been designed to target the viral membrane surface protein HA. The antibodies (Abs) that inhibit the binding of influenza viruses to host cells target the head domain, thereby conferring immunity to influenza (Chan, 2021).

The necessity for the reformulation of vaccines originates from antigenic drift, which can result in mismatches between vaccine strains and strains that are currently circulating. Such discrepancies are frequently linked with a reduction in the efficacy of the vaccine. In order to address this challenge and enhance vaccine efficacy, the World Health Organization has established biannual influenza vaccine composition meetings to try to select the correct vaccine seed viruses to match the circulating virus (Krammer and Palese, 2015; Yamayoshi and Kawaoka, 2019). To lower global morbidity and mortality, vaccines must be produced quickly in response to a new strain of the pandemic influenza virus (Krammer and Palese, 2015).

The development of next-generation seasonal, and in particular pandemic influenza vaccines, entails the consideration of novel techniques, in addition to only egg-, nanoparticle-, peptide-, and nucleic acid-based approaches (Cianci et al., 2020). An important but often overlooked point is that during a pandemic, it might be difficult to secure the necessary number of chickens, particularly if the pandemic originates from avian influenza, which may seriously affect the poultry stock potentially available. In contrast to the use of embryonated chicken eggs, the cell culture method or recombinant approaches obviates the necessity for an egg-adaptation process for vaccination strains, thereby facilitating the rapid expansion of production (Sekiya, 2021). To implement vaccination within a One Health framework, it is important to consider vaccinating animals—particularly poultry—against influenza viruses alongside humans, as this can help contain HPAI and reduce the risk of zoonotic transmission (Sims et al., 2016; Pushko, 2017). Achieving this would require the development of simple, affordable vaccines that can be easily and cost-effectively administered to animals (Pushko, 2017). Virus-like particles (VLPs) represent an attractive alternative platform to conventional influenza vaccine formulations due to their ability to mimic the native virus while lacking genetic material (Krammer and Palese, 2015; Mohsen and Bachmann, 2022). Furthermore, they can be produced in large quantities in *Escherichia coli*, which is a relatively simple and inexpensive process (Mohsen and Bachmann, 2022; Vogt, 2022). Recent research also demonstrates the versatility of VLPs in a variety of applications, including the treatment of infectious diseases (Krenger, 2024; Rothen, 2024), cancer (Josi, 2024a), and even in plant application (Josi, 2024b). Given the strong preclinical success of VLP-based vaccines targeting the extracellular domain of M (Neirynck et al., 1999; Jegerlehner et al., 2004; El Bakkouri et al., 2011), it is difficult to understand why its approach is not pursued more intensively.

Another type of cost-effective vaccines are live vaccines; these are developed to reproduce within an immunocompetent host, thereby achieving a balance between triggering a robust immune response and preventing the development of severe disease symptoms (Pollard and Bijker, 2021). Recent studies show that live attenuated influenza virus vaccines are a valuable tool for controlling and preventing swine influenza (Graaf-Rau, 2024). Furthermore, a number of influenza vaccines have been developed to combat HPAI, with the majority being inactivated whole-virus vaccines for use in hens (Nielsen, 2023).

Also, the development of mRNA vaccines gained a lot of traction in recent years regarding their use as a platform for influenza vaccines (Arevalo, 1979; Kackos, 2023). Furthermore, mRNA vaccines can have the potential to overcome many of the constraints that exist in vaccination systems such as the development of single formulation vaccines, the viability of incorporating more antigens, and enhanced efficacy and programmatic suitability (Leonard, 2024; Taaffe, 2024). The cost of goods and storage expenses, on the other hand, strongly argue against a One Health approach and, in fact, even preclude its use in humans in most parts of the world. Moreover, other expression techniques based on insect cells and baculovirus are utilized to create the recombinant HA vaccine. The advantages of this technique include a shorter manufacturing period, as there is no requirement for an adaptation process involving eggs, and the avoidance of undesirable mutations in the HA protein (Sekiya, 2021). In addition to costs, the tolerability of influenza vaccines is key in humans, while costs are key for animals; the broad application of RNA-based vaccines to combat seasonal flu in humans or flu in animals in general seems very unlikely.

### Vaccination in animals and the One Health approach

Vaccines offer protection in two distinct ways. Firstly, they reduce the risk of infection for individuals such as farmworkers and animals. Secondly, they limit the transmission and exposure of infectious diseases within the broader community (Oshansky, 2019). This dual effect highlights the relevance of vaccination within a One Health framework, where protecting animals not only safeguards their health and agricultural productivity but also reduces the risk of zoonotic transmission and pandemic emergence.

In the European Union (EU), two vaccines are currently authorized for use in chickens. Although these vaccines are not completely effective in stopping virus transmission, they remain the best available option for minimizing outbreaks at affordable costs. More candidates are at different stages of development. However, it underscores the need for vaccines specifically designed to meet the unique requirements of different poultry species and production systems (Authority, E. F. S, 2007; Nielsen, 2023). In areas where HPAI is endemic, vaccines are used to minimize the prevalence of HPAI viruses in the environment, thereby reducing human exposure, the risk of zoonotic and pandemic influenza, and the risk of severe disease in poultry. Given the predominance of the H5, H7, and H9 subtypes, vaccination that promotes protection against these viruses would be most beneficial (Pushko, 2017).

One challenge with today’s vaccines is that they do not completely protect animals from infection. For example, vaccinated chickens still excrete virus particles, known as silent spread. Also, the existence of interfering maternal antibodies is an important factor in a range of veterinary vaccination applications. Specifically, these antibodies have been shown to impede the immunization efficacy of newborn piglets against influenza, due to reduced replication of the vaccine strain (Chambers et al., 2016). The immune response to vaccination varies among different species and breeds of birds. For instance, antibodies generated in local breeds have been found to be lower than those in industrial chicken lines (Berg, 2008).

Monitoring of the vaccinated animals is therefore still necessary (Islam et al., 2023). In contrast to domestic animals, migratory birds are a highly mobile reservoir of AIV. Unlike the immunization against rabies in foxes, there is no technique to vaccinate or control AIV infection in the wild bird reservoir (Abdelwhab and Beer, 2024). Ideas are being considered for emergency vaccination of wild birds against HPAI (W.O.A.H. W. G. on W, 2023). However, infections in domestic reservoirs can be reduced through improved biosecurity measures, cost-effective culling, and the development and use of effective vaccines. Measures are needed to protect non-human mammals such as pigs and minks from infection with HPAI to prevent the spread of AIV from and to wild birds (Abdelwhab and Mettenleiter, 2023).

The implementation of vaccination against HPAI in poultry is constrained not only by biological efficacy but also by economic feasibility. A cost assessment of a preventive HPAI vaccination program in Austrian poultry farms demonstrated that total costs per vaccinated animal varied substantially, depending on farm type, regional risk, and surveillance requirements, with post-vaccination monitoring representing a major cost driver. Targeted, risk-based vaccination strategies were shown to be more cost-effective than nationwide blanket approaches (Marschik et al., 2025). These findings highlight a central One Health challenge: although animal vaccination can reduce viral circulation and zoonotic risk, financial and logistical barriers may limit its sustainable integration into coordinated animal and public health control strategies, particularly in resource-limited settings.

### Regulatory efforts and pandemic preparedness

The European Medicines Agency (EMA) has developed a distinctive authorization process for vaccines designed to combat potential influenza pandemics in humans. These vaccines typically comprise a strain of avian influenza, such as A/H5N1, which is uncommon in humans but represents a significant pandemic risk. Furthermore, additional strains are frequently tested to enhance understanding and preparedness. Although these vaccines may be authorized in advance, they are not distributed until a pandemic has occurred. Subsequently, the vaccine can be updated with the specific pandemic strain or zoonotic influenza strain that could cause a future flu pandemic and approved for use as the final version (European Medicines Agency (EMA), 2024). Importantly, rapid adaptation of vaccines to new strains is only possible with vaccine technologies that have undergone conventional registration, which is a very costly process, rendering it difficult to establish new vaccine technologies for the influenza virus.

One issue that currently exists is that the majority of vaccines available to the public or animals are directed against the HA antigen. Furthermore, the efficacy of the vaccination is determined by the HA titer, as there is a well-established correlation between the presence of antibodies against HA and the level of protection afforded by the vaccination. However, because HA is highly variable and subject to antigenic shift and drift, this narrow antigenic focus can reduce vaccine effectiveness against emerging or antigenically distinct strains. Other influenza virus surface proteins should therefore be considered as targets for vaccination. A significant number of vaccines currently in development focus on regions of the influenza virus (e.g., HA-stem (Guthmiller, 2022) and M2e (Calzas, 2024)) that exhibit less pronounced mutational dynamics than the HA head domains (cold spots). The objective is to develop a universal vaccine that, ideally, would not require annual refreshments. However, this would necessitate the creation of a novel framework for evaluating these novel vaccines, which would entail adapting the existing tests and a full registration process based on clinical cases rather than HA-specific antibodies (Yamayoshi and Kawaoka, 2019; Isakova-Sivak and Rudenko, 2024).

## Conclusion

In conclusion, to effectively reduce the risk of avian influenza infection in both humans and animals, it is important to both limit exposure and prevent the virus from spreading. We know that wild birds play a major role in the spread of HPAI. To achieve effective monitoring in accordance with the “One Health” concept, it is essential to foster multidisciplinary and cross-sectoral collaboration among medical, veterinary, and public health organizations. Furthermore, the exchange of information for early warning systems is of paramount importance (Warimwe et al., 2021; Duan et al., 2023). This underlines the need to learn more about the migration patterns and breeding grounds of waterfowl populations. Thus, the surveillance of wild birds at the intersection of migratory flyways to wintering areas would enrich epidemiological risk assessments and provide early warning of particular HPAI threats to poultry and possibly human health (Lycett, 1979; Adlhoch, 2024).

In addition, the prevention of the spread of H5N1 viruses from fur farms, mixed poultry-fur farms, and farms with both poultry and pigs to humans through enhanced biosecurity implementation in affected areas can be an effective way to prevent the outbreak of a pandemic (Horimoto and Kawaoka, 2005; Islam et al., 2023; Adlhoch, 2024). Access to rapid, cost-effective diagnostic tools is essential to screen animals and humans for early detection of the disease (Branda et al., 2024). Another objective is to maintain close cooperation between the animal health and human health sectors and the occupational health and safety authorities (Adlhoch, 2024).

To adequately prepare for future influenza pandemics, it is imperative that global and local efforts are coordinated. This should include the maintenance of antiviral stockpiles, the strengthening of supply chains for personal protective equipment, and the improvement of the detection of epidemiological, clinical, and laboratory signals through collaboration among veterinarians, healthcare workers, and laboratories (Kojima, 2024). However, these measures alone cannot guarantee the complete prevention of infection. The addition of vaccination programs for both humans and animals could further reduce the risk of infection and limit transmission (Food and Agriculture Organization of the United Nations, 2008). The combination of vaccination with surveillance is important, as it enables tracking of the virus’s evolution and any potential antigenic alterations, including vaccine-driven mutations in the viral population (Adlhoch, 2024).

Since the introduction of vaccination in the 18th century, advances in human and animal immunology have transformed vaccine technology and delivery methods. Research on animal infections and immune responses has made a substantial contribution to disease control, with novel vaccine platforms being tested in veterinary animals prior to their use in humans. Veterinary vaccines are subjected to rigorous safety and efficacy testing in their intended hosts, thereby establishing a benchmark for vaccine production (Warimwe et al., 2021). The necessity of preventative measures is exemplified by programs such as BARDA’s National Pre-Pandemic Influenza Vaccine Stockpile (NPIVS), which was created in 2005. The NPIVS, which was designed to supply vaccinations to 20 million key workers during a pandemic, underscores the importance of developing vaccine stocks for influenza strains with pandemic potential (Oshansky, 2019). In order to address the burden of influenza mortality and morbidity, efforts are being made to foster innovation in vaccine development. This includes the development of universal vaccines with broader and hence longer-lasting protection, greater efficacy against severe disease, and shorter production times (World Health Organization, 2019). It is imperative that efforts are made to enhance human and animal surveillance, create low-cost laboratory procedures, boost electronic communication, and increase vaccine manufacturing and delivery capacity. In the absence of a heightened sense of urgency, the global community is at risk of being exposed to the next pandemic (Snacken et al., 1999). The resolution of these difficulties necessitates the integration of scientific innovation and legislative action to address interrelated concerns, including global climate change, animal conservation, economic stability, and public health (Prosser et al., 2023).
